# Retraction: Masiá, C. et al. Effect of *Lactobacillus rhamnosus* on Physicochemical Properties of Fermented Plant-Based Raw Materials. *Foods* 2020, *9*, 1182

**DOI:** 10.3390/foods9121782

**Published:** 2020-12-01

**Authors:** Carmen Masiá, Poul Erik Jensen, Patrizia Buldo

**Affiliations:** 1 Department of Food Science, University of Copenhagen, Rolighedsvej 26, 1958 Frederiksberg, Denmark; cala@food.ku.dk (C.M.); peje@food.ku.dk (P.E.J.); 2 Food Cultures and Enzymes, Plant Based Application Projects & Competences, Chr. Hansen A/S, Bøge Alle 10-12, 2970 Hørsholm, Denmark

The journal retracts the article [1], cited above, published in the journal, *Foods*. Following publication, the authors contacted the publisher regarding the incorrect description of the methods used for the two analyses performed.

Adhering to our procedures, an investigation was conducted by the Editor-in-Chief with the editorial office of *Foods* that confirmed that the methods used in the experimental work were not the same as the procedures described in the submitted paper. The extent of the error is significant and, therefore, in accordance with the journal’s procedural requirements, this article must be retracted.

The authors agreed to this retraction and apologize for any inconvenience caused.
